# Childhood vitiligo impacts emotional health of parents: a prospective, cross-sectional study of quality of life for primary caregivers

**DOI:** 10.1186/s41687-020-0186-2

**Published:** 2020-03-20

**Authors:** Gabriela Andrade, Sneha Rangu, Lauren Provini, Elana Putterman, Abigail Gauthier, Leslie Castelo-Soccio

**Affiliations:** 1 Department of Child and Adolescent Psychiatry and Behavioral Sciences, The Children’s Hospital of Philadelphia, Philadelphia, PA USA; 2 Department of Pediatrics, Section of Dermatology, The Children’s Hospital of Philadelphia, 3401 Civic Center Blvd, Wood Center 3335, Philadelphia, PA 19104 USA; 3 Drexel University College of Medicine, Philadelphia, PA USA; 4 University of Alabama School of Medicine, Birmingham, AL USA; 5 University of Pennsylvania, Perelman School of Medicine, Philadelphia, PA USA

**Keywords:** Skin differences, Pigmentary disorders, Quality of life, Vitiligo, Pediatric dermatology

## Abstract

**Background/objectives:**

Individuals with vitiligo have an increased risk of depression, anxiety, social isolation and detrimental effects on body image/self-esteem. However, assessments of quality of life (QoL) impact have not focused on caregivers of children with vitiligo. To address this, we determined the QoL impact in parents of children with vitiligo to assess the relationship between QoL parameters and disease duration, location, and severity.

**Methods:**

We performed a cross-sectional study involving 123 parents of children diagnosed with vitiligo for at least 3 months, and who presented to the pediatric dermatology clinic of a major United States children’s hospital. Parents completed a demographics survey, Quality of Life in a Child’s Chronic Disease Questionnaire (QLCCDQ) and Family Dermatology Life Quality Index (FDLQI) to assess QoL measures. The lower the QLCCDQ score and higher the FLDQI score, the more quality of life is impaired.

**Results:**

Subject age ranged from 20 to 57, and 13.9% received mental health intervention. QLCCDQ emotional domain scores were most impaired, and severity and location of disease impacted these scores. FDLQI scores decreased as children age, indicating overall parent wellbeing increased as children age.

**Conclusions:**

Childhood vitiligo has great emotional impact on the quality of life of caregivers. Recognizing this will enable dermatologist who primarily care for these patients to incorporate care giver specific interventions during clinical visits. Emotional domain scores for parents of children with vitiligo were the most impaired as much or more than of those seen in parents of children with chronic stable medical disease such as type 1 diabetes and asthma.

## Introduction

Vitiligo is an acquired, multifactorial, and progressive pigmentary disorder characterized by depigmented macules and patches [1]. The worldwide prevalence of vitiligo is estimated to be 0.5–2%, with 50% of cases presenting prior to the age of 20. Since the depigmented patches of vitiligo are highly visible and often unpredictable, the disease carries a high psychosocial burden for patients [2]. Examination of the psychosocial impact among patients with vitiligo has revealed increased risk of depression, anxiety, and social isolation as well as detrimental effects on body image and self-esteem [2, 3].

Quality of life (QoL) assessments serve as an important tool for measuring the psychosocial burden among patients with chronic disease. This has relevance for adherence to treatment and long-term disease outcomes. In the case of pediatric patients with chronic disease like psoriasis, alopecia areata and atopic dermatitis, this burden has been shown to also involve the lives of parents and other primary caregivers [4–7]. To date, in the study of vitiligo, assessments of QoL impact have focused on pediatric patients but not on their caregivers [2, 3, 8–10]. To address this knowledge gap, the purpose of this study was to examine the QoL impact in parents of children with vitiligo using two externally validated QoL tools, the Quality of Life in a Child’s Chronic Disease Questionnaire (QLCCDQ) and the Family Dermatology Life Quality Index (FDLQI) [11, 12]. In addition, we sought to assess the relationship between these QoL parameters and disease duration, location, and severity, with the ultimate goal of highlighting the psychosocial burden among caregivers of pediatric vitiligo patients.

## Materials and methods

### Participants and procedures

Participants were identified by conducting a search of the EPIC database using the Recruitment Enhancement Core for patients seen in the Dermatology Department from 1/1/2013–1/1/2018 who carried a diagnosis of vitiligo by ICD-9 or ICD-10 codes for a minimum of 3 months (*n* = 1084). Eligible participants were parents or legal guardians of these patients who were English-speaking and at least 18 years of age. These individuals were invited to participate in the study through an initial e-mail detailing the study, as well as a link to the study survey in REDCap, a secure web application for online surveys and databases. The survey was initially sent to 227 participants, with 123 completing the survey. This study was approved by the Institutional Review Board of the Children’s Hospital of Philadelphia (IRB number: 17–013915).

### Questionnaire completion

The initial section of the survey contained questions about demographic information, including current age of participant and current age of pediatric patient with vitiligo, as well as the child’s gender, age at time of initial vitiligo diagnosis, and duration of disease. This portion of the survey also addressed the clinical severity of the patient’s vitiligo asking parents to estimate percent body surface area (BSA) involvement (0%–25%, 25%–50%, 50%–75% and 75%–100%), the anatomic locations of the lesions (head/face/neck, trunk, arms (not including hands), hands, legs and genitalia) and the child’s background skin pigmentation (based on the Fitzpatrick scale). There are currently no standardized tools to calculate severity of vitiligo, and estimating BSA involvement was the optimal option for subjective estimation of severity. Finally, we asked whether the child or caregiver had ever undergone any mental health intervention, such as therapy or psychiatric medication.

Participants then proceeded to the second section of the survey, which contained two externally validated tools, the 15 question QLCCDQ and the 10 question FDLQI (see Appendix). The QLCCDQ is a general chronic illness QoL tool targeted towards parents of children with chronic disease [11]. The FDLQI is a more specific dermatologic disease QoL tool intended for family members of patients with chronic dermatoses [12].

### Statistical analysis

Spearman’s Rank Correlations were used to examine the association between QLCCDQ and FDLQI scores and the clinical severity of the vitiligo as determined through disease duration, anatomic location of lesions, the approximate affected BSA, and the age of child. Statistical significance was defined as *p* < 0.05. Statistical analyses were conducted using [STATA/SE 14] and [SAS OnDemand for Academics/Version 3.7].

## Results

### Patient and parent characteristics

A total of 123 subjects were included in this study (Table 1). The mean age for children with vitiligo at time of diagnosis was 6.1 years (range: 1 to 15) and male to female ratio was 1.1:1. Children had varying disease severity, represented by percent BSA with vitiligo lesions: 81 (65.9%) of patients had 0–25%, 26 (21.1%) had 25–50%, 10 (8.1%) had 50–75% and 6 (4.9%) had 75–100%. Twenty (16.3%) children received mental health intervention including therapy or psychiatric medication.

**Table 1:** Characteristics of patient participants with vitiligo

Characteristic	Mean (SD)	Range
**Age at diagnosis, years**	6.1 ± 3.5	1–15
**Duration of disease, months**	69.6 ± 43.6	4–192
	**Frequency**	
**Area of involvement**		
**0%–25%**	65.9% (*n* = 81)	
**25%–50%**	21.1% (*n* = 26)	
**50%–75%**	8.1% (n = 10)	
**75%–100%**	4.9% (*n* = 6)	
**Anatomical parts of involvement**		
**Head/Face/Neck**	59.3% (*n* = 73)	
**Trunk**	49.6% (*n* = 61)	
**Arms (not including hands)**	41.5% (*n* = 51)	
**Hands**	36.6% (*n* = 45)	
**Legs**	62.6% (*n* = 77)	
**Genitalia**	33.3% (*n* = 41)	
**Skin type**^a^		
**I**	0.8% (*n* = 1)	
**II**	11.5% (*n* = 14)	
**III**	30.3% (*n* = 37)	
**IV**	37.7% (*n* = 46)	
**V**	13.1% (*n* = 16)	
**VI**	6.6% (*n* = 8)	
**% received mental health intervention (therapy or psychiatric medication)**	16.3% (*n* = 20)	

^a^Based on Fitzpatrick scale

The mean age for parents was 43.4 ± 7.0 years (range: 20 to 57 years) (*n* = 123). Seventeen (13.9%) parents received mental health intervention including therapy or psychiatric medication.

### FDLQI and QLCCDQ scores

Descriptive results for the FDLQI and QLCCDQ completed by parent participants are shown in Table 2, including the FDLQI overall score and both the QLCCDQ overall score and individual domain sub-scores, stratified by disease severity (percent BSA with vitiligo lesions).

**Table 2:** Summary of QoL survey results by % of area of involvement in vitiligo participants

Patient/parent grouping (area of involvement)	N (%) for each survey (FLDQI and QLCCDQ)	Mean FDLQI overall score ± SD	Mean QLCCDQ per-item score ± SD	Mean QLCCDQ social domain score ± SD	Mean QLCCDQ occupational roles domain score ± SD	Mean QLCCDQ symptom domain score ± SD	Mean QLCCDQ emotional roles domain ± SD score	Mean QLCCDQ family perception domain score ± SD
**0–25%**	78 (66.1%, FLDQI); 77 (65.3%, QLCCDQ)	12.3 ± 2.7	6.1 ± 1.2	6.7 ± 1.2	6.5 ± 1.2	6.0 ± 1.5	5.4 ± 1.5	6.6 ± 1.2
**25–50%**	26 (22.0% FLDQI; 22.2% QLCCDQ)	14.0 ± 4.3	5.7 ± 1.2	6.6 ± 1.1	6.3 ± 1.5	5.0 ± 1.4	4.8 ± 1.6	6.7 ± 1.1
**50–75%**	8 (6.8% for both FLDQI and QLCCDQ)	15.3 ± 3.5	5.7 ± 0.7	6.9 ± 0.3	6.7 ± 0.4	4.7 ± 1.6	4.1 ± 2.0	6.9 ± 0.2
**75–100%**	6 (5.1% for both FLDQI and QLCCDQ)	15.8 ± 6.5	5.3 ± 1.0	6.4 ± 1.0	5.7 ± 1.7	4.7 ± 1.4	4.2 ± 1.1	6.3 ± 1.1
**Overall**	118 (FLDQI); 117 (QLCCDQ)	13.1 ± 3.5	5.9 ± 1.1	6.6 ± 1.3	6.4 ± 1.5	5.6 ± 1.6	5.1 ± 1.6	6.6 ± 1.1

As shown in Table 3, a statistically significant positive correlation was found between the BSA of involvement and the FDLQI overall score; this corresponded with a statistically significant negative correlation between the BSA involved and both the QLCCDQ overall score as well as the QLCCDQ emotional domain-specific score. While the total disease duration had a positive correlation with the FDLQI overall score and a negative correlation with the QLCCDQ overall score, these were not statistically significant. Finally, patient age was found to have a statistically significant negative correlation with the FDLQI overall score. Patient age was positively correlated with both the QLCCDQ overall and emotional domain-specific scores, though these relationships were not statistically significant.

**Table 3:** Correlations (and *p*-value) between parent QoL measures and % of area involvement, duration of disease and age at diagnosis of child with vitiligo

	FDLQI overall score	QLCCDQ per-item score	QLCCDQ emotional domain score
**Area of involvement**	0.26 (0.039)^*^	−0.33 (0.0003)^*^	−0.30 (0.0095)^*^
**Duration of disease**	0.048 (0.61)	−0.012 (0.90)	0.072 (0.93)
**Age of child**	−0.19 (0.036)^*^	0.10 (0.27)	0.20 (0.53)

^*^denotes statistical significance (*p* < 0.05)

## Discussion

In this study, we describe and evaluate QoL in parents of patients with vitiligo. FDLQI scores (12.3–15.8, Table 2) were lower than those reported in parents of children with atopic dermatitis (13.6–17), but higher than the range of scores reported in psoriasis (8.8) and alopecia areata patients (6.5) [6, 13–15]. On average, QLCCDQ emotional domain scores (5.1) were slightly higher than those reported in parents of children with stable, chronic medical disease (4.5) such as type 1 diabetes and asthma [11].

Previous work examining the impact of vitiligo on QoL has shown patients with a BSA more than 25% have exhibited symptoms of self-consciousness, difficulty with friendships and schoolwork [16]. However, there is no information on parental QoL in regards to area of involvement of their child’s vitiligo. Our study adds to the findings of earlier QoL studies showing that overall parental QoL decreases with increasing area of involvement through both the FDLQI (*r* = 0.26, *p* = 0.0039, Table 3) and QLCCDQ surveys (*r* = − 0.33, *p* = 0.0003, Table 3). Amongst all QLCCDQ domains, the emotional domain showed the greatest QoL impairment on average (5.1, Table 2). It is important to note that children with high BSA involvement may require more involvement of parents’ time in the treatment.

We found no significant correlation between duration of disease and overall parental QoL. These findings may suggest that the initial psychosocial and functional impact at time of diagnosis is sustained throughout the duration of disease but could also be due to natural waxing and waning of disease. However, emotional QoL significantly correlated with patients who have vitiligo on their hands and legs (*r* = − 0.10, *p* = 0.0068; *r* = − 0.17, *p* = 0.04). Other parts, such as the trunk, arms (not including hands) genitalia, face/neck/head showed no significant correlation. One possible explanation for these anatomic correlations is that patients may find it easier to keep patches on trunk, genitalia, head, and neck covered with clothing, whereas the hands and legs may be more difficult to conceal. Interestingly we did not find a correlation on the face/neck/head. Perhaps here to it is easier to conceal with makeup/cosmetics.

Patient age did not correlate with QLCCDQ per-item scores, but there was a slight correlation between patient age and FLDQI overall scores (*r* = − 0.19, *p* = 0.036, Table 3). A relationship between patient age and parental scores has been previously described in a study done on the QoL in parents of children with alopecia areata [15]. The alopecia areata study suggests that overall parental QoL may decrease as children age through a slight negative correlation between QQLCDQ per-item scores, but not FLDQI overall scores. In contrast, our study suggests that parental QoL is worse in younger patients, and that QoL increases as children age. Thus, the opposing results from our study and the alopecia areata study exemplify that while QoL may differ as children age depending on the skin condition, QoL may be more closely related to area of involvement/severity of disease than either age of the child or duration of disease.

## Conclusions

This study is the first to describe QoL in parents of children with vitiligo in the United States. The only other study that described QoL in parents of children with vitiligo previously was done in China [10]. This study used different measures, the Self-rated Health Measurement scale (SRHMS) and the Dermatitis Family Impact Questionnaire (DFIQ), and showed that parents of children with vitiligo have significant psychological problems and poor QoL compared to parents of unaffected children. Similarly, our study shows a decreased QoL in parents of children with vitiligo in the United States. Our use of the QLCCDQ offers a more targeted investigation than using only the FDLQI might allow, or the measures used in the study done in China. The QLCCDQ is developed for parents, while the DFIQ and FLDQI is intended for broad use in any first-degree family member of affected patients [12]. This disparity in the surveys’ intended uses may partly explain why FLDQI and QLCCDQ scores correlated moderately in our study (*r* = − 0.62). Additionally, the QLCCDQ allows us to evaluate particular components of overall QoL through its domain-based approach. While our sample size is limited in size, it is highly representative of the greater population patients with vitiligo and their parents. Data for this study was collected in a clinical setting free of selection bias that may exist when a convenience sample is used, such as participants who are recruited through national foundation or support groups where many severe cases may exist.

Moreover, primary caregivers serve as the primary liaison between physician and patients, which creates a need for caregiver-oriented patient care. The clinical implications of our study’s results highlight the intersection between psychology and dermatology. First, it is important for providers to be aware that area of involvement/severity predicts parental QoL impairment but only to some extent, so that we are careful not to neglect counseling and education that is warranted in families facing milder disease in addition to those dealing with severe disease. Second, since the greatest impairment in parents’ QoL is emotional, providers should tailor counseling accordingly. Adjustments during clinic visits can be made by asking parents how the disease has affected them and providing outside resources such as support group information or additional educational resources. Lastly, the finding that parent overall wellbeing increases as children age can be used to give some relief to parents, while setting realistic expectations, for long-term prognosis and course of the disease.

Further research on the QoL in parents of children with chronic diseases is needed, as caregiver QoL and perception of a child’s symptoms is vital in treating children [17, 18]. Given that emotional QoL scores were mostly impacted by vitiligo, stigma focused QoL instruments and surveys designed for use in parents or patients dealing with vitiligo can advance the assessment of the psychosocial and functional challenges that present in this visible skin disease.

## Limitations

Our study is limited by its small sample size and cross-sectional design. Those who responded might be those who are highly impacted, thus introducing a possible recruitment bias. Factors we could not or did not account for that may have affected parental QoL include social support and whether the child was improving or worsening at the time of survey completion. We were not able to determine status of disease through retrospective review, given that vitiligo can flare and remit in the time interval between visits. Lastly, subjective reporting of BSA may have led to unreliable estimations for disease severity.

## Data Availability

All data and materials were made available to all authors and all consent to publication of work.

## Abbreviations

BSA: Body Surface Area
CHOP: Children’s Hospital of Philadelphia
FDLQI: Family Dermatology Life Quality Index
IRB: Institutional Review Board
QLCCDQ: Quality of Life in a Child’s Chronic Disease Questionnaire
QoL: Quality of Life
